# Review of Integrated Optical Biosensors for Point-of-Care Applications

**DOI:** 10.3390/bios10120209

**Published:** 2020-12-18

**Authors:** Yung-Tsan Chen, Ya-Chu Lee, Yao-Hsuan Lai, Jin-Chun Lim, Nien-Tsu Huang, Chih-Ting Lin, Jian-Jang Huang

**Affiliations:** 1 Graduate Institute of Photonics and Optoelectronics, National Taiwan University, No. 1, Sec. 4, Roosevelt Road, Taipei 106, Taiwan; d03941013@ntu.edu.tw (Y.-T.C.); r08941047@ntu.edu.tw (Y.-C.L.); r07941068@ntu.edu.tw (Y.-H.L.); r07941106@ntu.edu.tw (J.-C.L.); 2 Department of Electrical Engineering, National Taiwan University, No. 1, Sec. 4, Roosevelt Road, Taipei 106, Taiwan; nthuang@ntu.edu.tw (N.-T.H.); timlin@ntu.edu.tw (C.-T.L.); 3 Graduate Institute of Biomedical Electronics and Bioinformatics, National Taiwan University, No. 1, Sec. 4, Roosevelt Road, Taipei 106, Taiwan; 4 Graduate Institute of Electronics Engineering, National Taiwan University, No. 1, Sec. 4, Roosevelt Road, Taipei 106, Taiwan

**Keywords:** optical biosensors, point-of-care, integration

## Abstract

This article reviews optical biosensors and their integration with microfluidic channels. The integrated biosensors have the advantages of higher accuracy and sensitivity because they can simultaneously monitor two or more parameters. They can further incorporate many functionalities such as electrical control and signal readout monolithically in a single semiconductor chip, making them ideal candidates for point-of-care testing. In this article, we discuss the applications by specifically looking into point-of-care testing (POCT) using integrated optical sensors. The requirement and future perspective of integrated optical biosensors for POC is addressed.

## 1. Introduction

A biosensor is an analytical device that is composed of a bioreceptor, a transducer, and a signal processor for detecting biological substances and monitoring biological interactions. The transduction methods for biosensing applications are mainly based on optical, physical, chemical, electrochemical, or mechanical properties of the functional biorecognition materials (e.g., enzyme, antibody, aptamer, and DNAzyme) [1,2,3,4]. The sensitivity and selectivity are two critical parameters in the development and application of biosensors. Among various types of sensors, optical biosensors offer great advantages over conventional analytical techniques because they enable direct, real-time, and label-free detection of many biological and chemical substances. Their advantages include high specificity, sensitivity, small size, and cost-effectiveness. The non-invasive optical detections have developed, in the past decade, from fluorescent spectroscopy to grating, SPR (surface plasmon resonance), SERS (surface-enhanced Raman scattering), GMR (guided mode resonance), etc. Fundamental light emission or light–matter interaction, such as fluorescent light emission from target analyte, optical diffraction due to light device structure interactions, and optical resonance within an optical cavity or designated structure, is employed by these optical sensing methods. 

With the advance of clinical diagnostics, modern biosensing gradually evolves from off-site laboratory tests to near the patient on-site diagnosis [5,6,7]. The testing modality, referred to as point-of-care testing (POCT), emphasizes on less skilled labor involvement, easy-to-use, rapid diagnosis, and compact size at an affordable price [5,6,7,8]. Currently, POCT is available for a series of analyses by shrinking the instruments for the laboratory testing in size. For example, POCT is found for pregnancy testing, cardiometabolic testing, glucose testing, infectious disease testing (such as HIV, respiratory infection, and sexually transmitted diseases) and numerous other applications [8,9,10,11,12,13]. The above devices for point-of-care (POC) and those in development rely on various technologies. Integrated biosensors have the advantages of combining different test functionalities, miniaturization by embedding different sensor components, and integration with semiconductor fabrication process. For example, the lab-on-a-chip combines several analyses that are usually carried out in the laboratory into a single chip [14,15]. It represents a significant contribution toward POCT. The integration can be extended to other devices with different detection principles. Optical sensors integrated with microfluidic channels, electrical charge sensing, and mechanical sensing are particularly promising for next-generation POCT [16,17,18,19,20,21,22].

The present article provides an overview of recent advances in optical biosensors and their integration with microfluidic channels. The integrated biosensors provide two areas of advantage. First of all, by simultaneously monitoring two or more parameters, the accuracy and sensitivity of detecting target analytes are improved. Second, the integration, most of the time, shrinks the overall system by incorporating various components (including light source, transducer, detector, and reader) in the biosensor [23,24,25]. The sensor can further integrate many functionalities monolithically in a single chip by the semiconductor process. We hope the overview provides some hints for the interested readers to extend the applications of optical biosensors by considering many different sensing mechanisms. Finally, we discuss the applications by specifically looking into POCT using integrated optical sensors. The requirement and future perspective of integrated optical biosensors for POC is then addressed.

This article first classifies optical biosensors that are mostly discussed in the literature. These biosensors are fluorescence-based biosensors, SERS-based biosensors, grating and photonic crystal (PC) biosensors, GMR biosensors, and plasmon-based biosensors. The principle, design methodology, and biosensing applications are briefly introduced to provide readers the fundamental understanding of an optical biosensor. Although fiber based biosensing is also one of the most important sensing technologies, the fundamental principles usually derive from the aforementioned sensing approaches. Thus, fiber-based sensors are not covered in this paper. Interested readers can refer to some of the excellent reviews in this topic [26,27]. Because biosensing based on microfluidic platform allows simultaneous detection of physical, chemical, and mechanical properties of the solution, in Section 3, we review the integration of fluidic platforms with various optical biosensing technologies. Since not all integrated biosensors can be readily available for POCT, the requirement and classification of recent development are introduced in Session 4, along with a tabulated review of integrated biosensors for POCT.

## 2. Classification of Optical Biosensors—Overview of the Principle and Applications

### 2.1. Fluorescence-Based Biosensors

**Figure 1:**
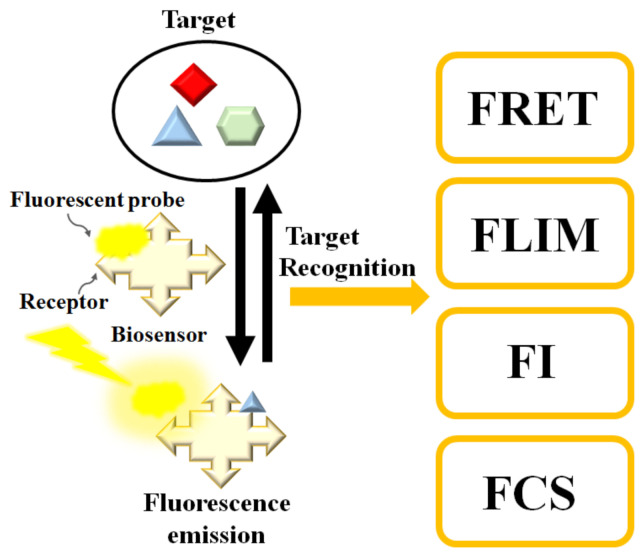
Schematic diagram of the fluorescence-based biosensor. Target analyte can be determined by FRET (Förster resonance energy transfer), FLIM (fluorescence lifetime imaging), FI (changes in fluorescence intensity), or FCS (fluorescence correlation spectroscopy).

Fluorescence biosensors detect the concentration, location, and dynamics of biomolecules on the basis of the fluorescent phenomenon that occurs when electromagnetic radiation is absorbed by fluorophores or fluorescently labeled molecules so that the energy is converted into fluorescence emission (see Figure 1) [28,29,30]. A fluorescence biosensor includes the excitation light source (LEDs (light-emitting diodes), lasers), fluorophore molecules that label target biomolecules, and a photodetector that records the fluorescence intensity and spectrum. The fluorophore molecules can be small molecules, proteins, or quantum dots that are used to label proteins, nucleic acids, or lipids [31]. Generally, the excitation signal is biorecognized by the following techniques [32]: (1) FRET (Förster resonance energy transfer), (2) FLIM (fluorescence lifetime imaging), (3) FCS (fluorescence correlation spectroscopy), and (4) FI (changes in fluorescence intensity). In the next session, we review fluorescence-based biosensors integrated with microfluidic channels.

### 2.2. Surface-Enhanced Raman Scattering-Based Biosensors

**Figure 2:**
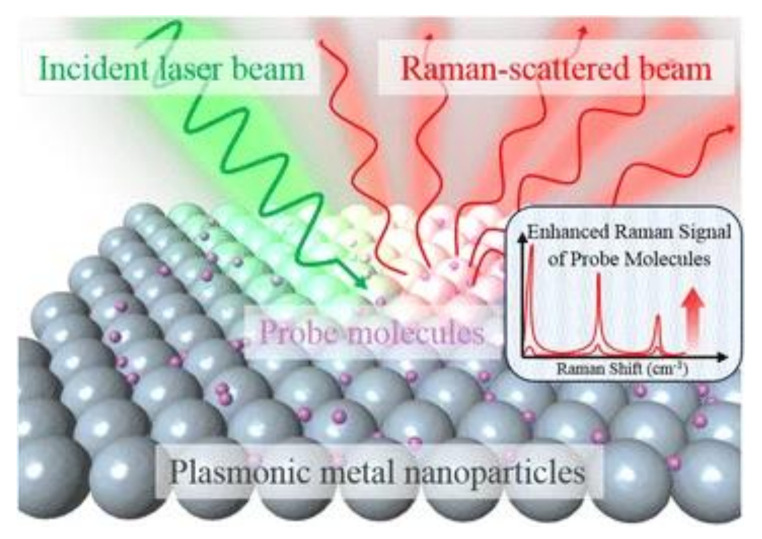
Schematic diagram of the SERS (surface-enhanced Raman scattering) process using metal nanoparticles to enhance the sensitivity [39]. Reproduced with permission from [39]. Copyright (2016) Springer Open.

Raman scattering is an inelastic process when the kinetic energy of the incident photons is increased or decreased by interacting with molecular vibrations or rotations, phonons, or other excitations. The spectrum of the scattered photons indicates energy changes that are specific to the vibrational or rotational transitions modes of the molecular structure, which the biosensing of biomaterials is based upon. Because spontaneous (normal) Raman scattering is typically very weak, surface-enhanced Raman spectroscopy or surface-enhanced Raman scattering employs surface-sensitive techniques to enhance Raman scattering by molecules adsorbed on a specific medium or interface to improve the sensitivity [33,34]. The enhancement factors for SERS, as compared to normal Raman scattering, are attributed to two mechanisms—an electromagnetic mechanism and a chemical mechanism [35,36,37,38]. The electromagnetic enhancement is generally considered as a major factor of SERS enhancement. The effect of wavelength shift is contributed from the excitation of localized surface plasmon resonance (LSPR) [39] (see Figure 2). It is regarded as the oscillation of conduction electrons in noble metal nanoparticles, sharp metal tips, or roughened metal surfaces. LSPR is a short-range detection in the vicinity of the metal surface (0–5 nm of the surface) [39,40]. The chemical mechanism depends not only on the substrate but also analyte molecules. It involves several different transitions/processes, which charge transfer between the energy levels of analyte molecules, with the fermi level of the substrate metal surface playing a critical role [41]. 

Recently, the studies on SERS biosensing have gradually migrated from probing single location of the plasmonic structure to detecting many spots through bioimaging with SERS tag [42,43]. In order to improve the electromagnetic enhancement factor, researchers have demonstrated various fabrication methods, such as adjusting the morphology, dielectric property, and interparticle distance of the plasmonic nanostructures [44,45]. 

### 2.3. Photonic Crystal-Based Biosensors

**Figure 3:**
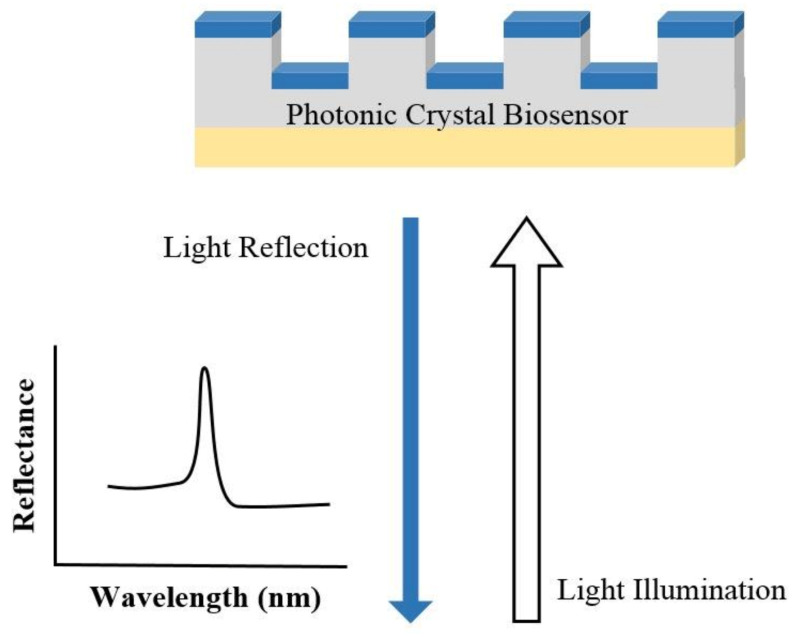
Illustration of the sensing mechanism of a photonic crystal (PC) biosensor.

In PC structure, optical resonant modes or photonic bandgaps are created due to the periodic arrays of the refractive index. The periodic structure can be a Bragg reflector, one-dimensional (1D) slabs, or two-dimensional (2D) PC. An example of a PC biosensor is shown in Figure 3, where the resonant modes of the reflection spectrum are understood from the Bragg’s law of diffraction. Since the resonant wavelength is sensitive to the refractive index of the materials on the PC structure, PC can be used for biosensing [46,47]. The narrow (<1 nm) bandwidth and high (≈95–100%) reflectivity resonances enable PC biosensors for detecting small molecules, virus particles, DNA microarrays, and live cells [46,47,48,49,50,51,52].

Recent advances in PC biosensors are focused on optimizing the instrumentation and device structures. For example, PC fluorescence enhancement was utilized for high-sensitivity multiplexed cancer biomarker detection [53]. Moreover, on the basis of the PC surface mode detection, researchers have developed label-free flow multiplex sensing of cancer biomarkers [54]. 

### 2.4. Guided Mode Resonance-Based Biosensor

**Figure 4:**
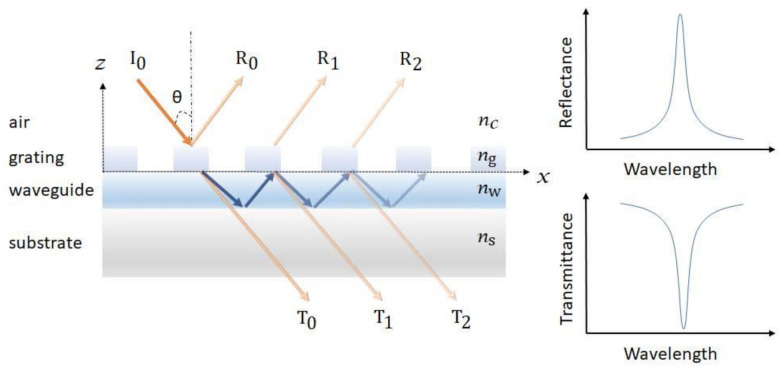
Schematic of a guided mode resonance (GMR) sensor. The resonance can be observed from both the reflectance and transmittance spectra.

GMR is a resonance phenomenon occurred in the structure with an optical diffraction element and a waveguide layer. As shown in Figure 4, the guided modes are excited in the optical waveguide and are extracted to the air (reflectance R_0_, R_1_, R_2…_ and transmittance T_0_, T_1_, T_2_..) by interacting with the diffraction structure, resulting in a very narrow reflectance or transmittance resonant bandwidth [55,56,57,58,59]. The resonant peak is highly sensitive to the changes in surrounding refractive index, which is used in determining target analytes and the corresponding concentrations [58].

Recent advances in GMR sensor focus on the redesign of surface diffraction structure and improvement of sensitivity [60]. A “grating–waveguide” GMR sensor with coupled cross-stacked gratings was demonstrated to achieve a high wavelength and angular shift FOM (figure-of-merit) [61]. Moreover, an ultra-sensitive refractive index sensor employing phase detection in a GMR structure was also reported by incorporating the GMR structure in to a Mach–Zehnder interferometer. The researchers achieved a minimum phase shift of (1.94 × 10^−3^) π that corresponded to a refractive index change of 3.43 × 10^−7^ [58].

### 2.5. Plasmon-Based Biosensors

The coupling of light with continuous metallic films (SPR (surface plasmon resonance)) or metallic nanoparticles (LSPR) produces strong confinement of electromagnetic field intensity. The confined field enhances the interaction between light and target molecules, resulting in the increase of sensitivity. 

**Figure 5:**
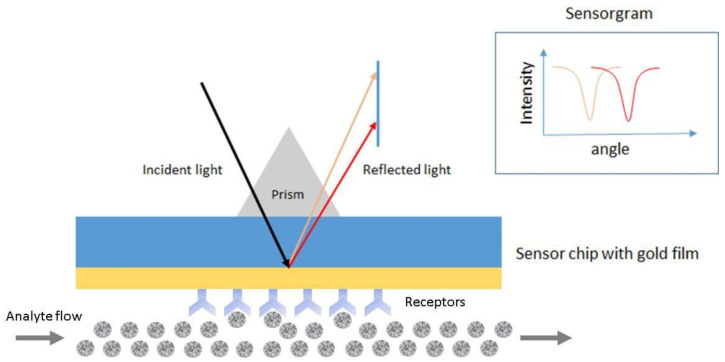
Schematic configuration of an surface plasmon resonance (SPR) biosensor.

SPR is the resonant oscillation of conduction electrons at the interface between negative and positive permittivity material stimulated by incident light [62,63]. Figure 5 shows an example of a schematic configuration of an SPR biosensor. The incident light is totally internally reflected from the prism. The evanescent waves outside the prism interact with the plasma waves on the surface of the metal (typically Au or Ag) and induce plasmon resonance. SPR can be observed from the angle of minimum reflection (angle of maximum absorption), which is very sensitive to the refractive index of the biomaterial applied on the metal surface [63,64]. The method can be used to detect molecular adsorption, such as polymers, DNA, proteins, or molecular interactions [65,66,67]. 

LSPR is the plasmonic effect that occurs when the conducting electrons within the nanoparticles are excited and oscillated with the incident light, a phenomenon that is mentioned in Section 2.2. LSPR-based biosensor monitors the changes of plasmon frequency with the local refractive index of the target analyte in the near field of nanoparticles. The nano-sized sensor has the advantage of portability and miniaturization, as compared to the planar-type SPR sensor.

Up until now, it has still been challenging for plasmon-based biosensors to detect small molecules or very low concentration of analytes. To improve LOD (limit-of-detection), recent effects on the development of SPR biosensing have focused on choosing proper receptors and calibration strategy, while for LSPR, a combination of fluorescent quantum dots with plasmonic metal nanoparticles has been exploited [68]. In addition, Ewa Gorodkiewicz et al. has an excellent review paper on recent publications on SPR biosensors between 2016 and 2018 [69]. Depending on the maturity of development, the authors categorized SPR biosensors into five stages ranging from simple marker detection to clinical application of the previously developed biosensors. Instrumental solutions and details of biosensor construction were analyzed by including chips, receptors, and linkers.

## 3. Methods of Integration with Optical Biosensors

Optical biosensors embedded within the microfluidic channel is one of the most common approaches for biosensing applications. In this section, optical biosensors with microfluidic channels embedded in the sensor substrate are addressed. The main focus of the discussion is the microfluidics and how various optical biosensors are integrated.

### 3.1. Microfluidics with Fluorescence Sensing

Currently, major advancements in FRET, FLIM, FCS, and FI have revolutionized biology by increasing the resolution of optical microscopy, which provides non-invasive observation methods. Further integration with microfluidics allows precise manipulation of cells and proteins. Here, we describe some recent developments in these fields.

#### 3.1.1. Fluorescence Resonance Energy Transfer

FRET relies on the transfer of excitation energy of a donor fluorophore to a nearby acceptor fluorophore through long-range dipole–dipole interactions. This occurs when the separating distance is within several nanometers. This technique has been widely used in the study of protein and DNA molecules [70,71]. More recently, Srisa-Art et al. used a microfluidic platform to perform binding assays and kinetics between streptavidin and biotin via FRET [72].

#### 3.1.2. Fluorescence Lifetime Imaging

**Figure 6:**
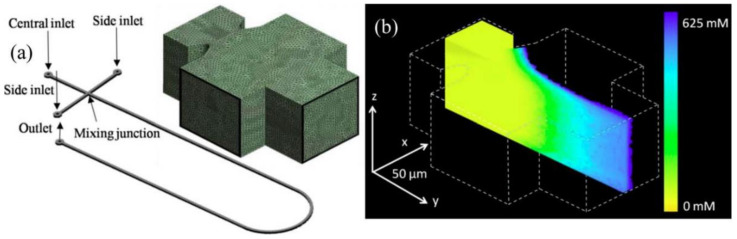
(**a**) Schematic of channel layout and (inset) computational fluid dynamics simulation (CFD) mesh of mixing junction. (**b**) 3D (three-dimensional) CFD simulation result of the mixing junction. Reproduced with permission from [73]. Copyright (2008) OSA publishing.

FLIM is a fluorescence imaging technique where the contrast is determined by differences in the excited state decay rate from a fluorescent sample. Robinson et al. demonstrated three-dimensional molecular imaging in a microfluidic mixer using fluorescent quenching [73] (Figure 6). More recent papers utilized FLIM for protein tracking and for the characterization of enhanced green fluorescent protein (EGFP) photoconversion [74]. 

#### 3.1.3. Fluorescence Correlation Spectroscopy

**Figure 7:**
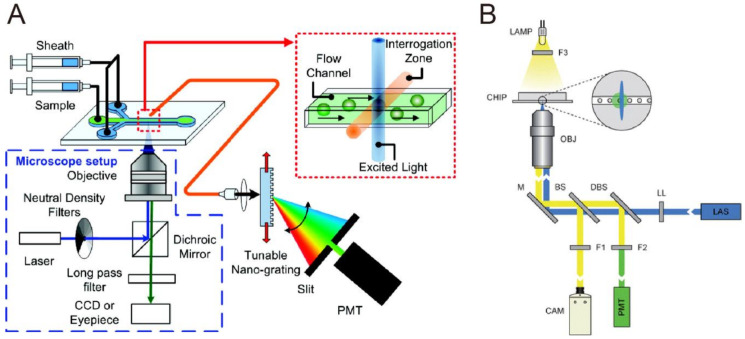
(**A**) Schematic of the microfluidic multispectral flow cytometry (MMFC) system. Reproduced with permission from [76]. Copyright (2010) ACS publications. (**B**) Schematic representation of the fluorescence-activated droplet sorting (FADS) system. Reproduced with permission from [77]. Copyright (2009) Royal Society of Chemistry.

An integrated microfluidic system can be used for high-sensitivity detection, as well as for sorting of fluorescent cells and particles [75]. Huang et al. integrated tunable nano-grating with a microfluidic channel to discriminate between green fluorescent protein (GFP) and calcein AM-stained HeyA8 cells (Figure 7A). Their system was able to differentiate between in-flow emission sources with only 5 nm peak-to-peak spectral differences and a significant intensity overlap at a confidence level as high as 85% [76]. Another example was demonstrated by Baret et al., who used the oil–water interface to encapsulate fluorescent-labeled single cells in the microdroplet and utilized fluorescent-activated cell sorting manner to separate two different strains of *Escherichia coli* bacteria on the basis of enzymatic activity (Figure 7B) [77]. The sorting rate can be ≈300 droplet s^−1^ with only 1/10,000 false positive error.

#### 3.1.4. Fluorescence Intensity

**Figure 8:**
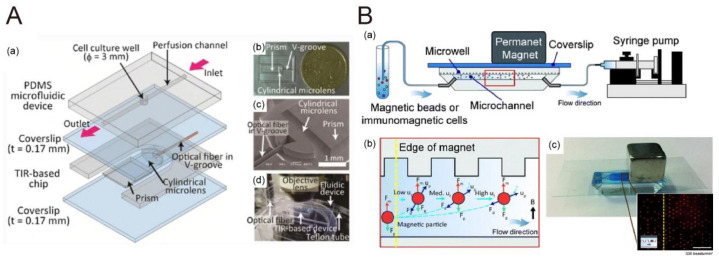
(**A**) The microfluidic device with on-chip total internal reflection fluorescence microscopy (TIRFM). Reproduced with permission from [78]. Copyright (2012) Springer. (**B**) A microfluidic microwell device for trapping fluorescent labeled single cells. Reproduced with permission from [79]. Copyright (2018) Springer.

Yokokawa et al. integrated microfluidic channels with a total internal reflection (TIR) chip to observe fluorescent beads and insulin granules (Figure 8A). The SNR (signal-to-noise ratio) obtained was comparable to that of commercial TIRMF systems and was twice that of epifluorescence microscopy (EPIFM) [78]. Another example is demonstrated by Lai et al. and Huang et al. used optical fluorescence imagery to count cells trapped within microwell arrays, which allowed for automation of image processing (Figure 8B) [79].

Another example of integrating microfluidics with fluorescent sensing was demonstrated by Kumar et al., who developed a 3D-tapered metal-insulator-metal (MIM) waveguide device to greatly enhance fluorescence signal of biomolecules on the basis of surface plasmon polarization (SPP). By integrating microchannel to guide targeted biomolecules, the device allows for the detection of 10 pM immunoglobulin G (IgG), which can potentially be applied for complex molecules or even single-molecule detection [80].

### 3.2. Microfluidics with SERS

#### 3.2.1. Advantages of Integrating Microfluidics with Surface-Enhanced Raman Spectroscopy 

SERS has emerged as a powerful analytical technique in recent years, allowing rapid, label-free, non-invasive, and highly specific cell, microorganism, or chemical species or biomolecular identification. Although SERS is a highly specific detection method and is well suited for chemical issues, using SERS for practical sensing is still challenging as it requires tedious sample manipulation and sophisticated instrumentation due to the low sensitivity and low reproducibility of spectra [81]. SERS-based optofluidic integrated with microfluidics, which could deal with constrained small volume fluids, enabling fast and flexible sample treatments in a microchip, has the potential to improve the utility of the SERS effect in practical applications [82]. On-chip SERS detection with well-controlled flow conditions yields more reproducible results, and several problems—for example, variable mixing times, variable scattering geometries, localized heating, and photo-dissociation—could be effectively solved. Integrating with microfluidics provides more consistent geometries and heat dissipation properties, which benefits photosensitive or heat-sensitive analytes [83].

#### 3.2.2. Microfluidics with Metallic Nanostructures for SERS Sensing

**Figure 9:**
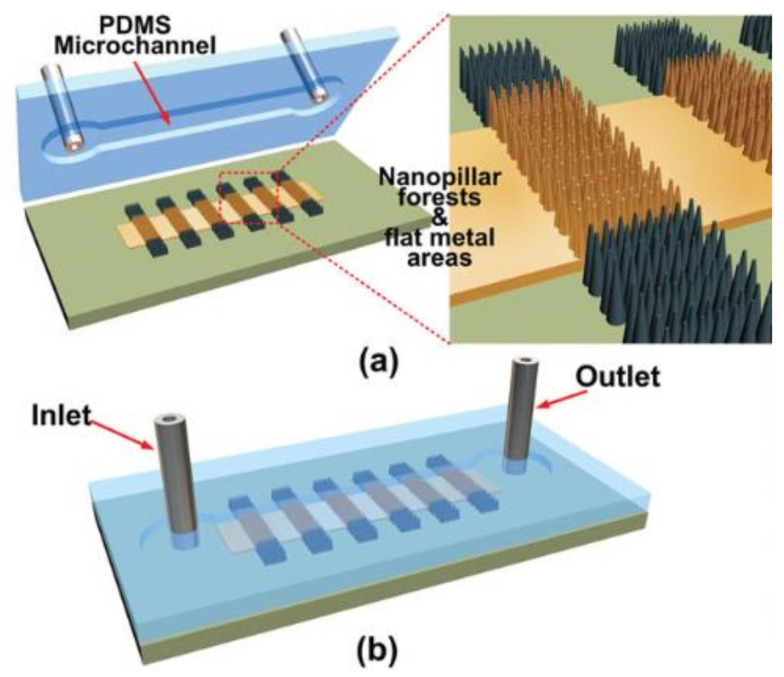
Schematic structures of the microfluidic SERS sensor: (**a**) schematic details on the SERS-active substrate with patterned nanopillar forests and flat metal areas for self-detection. (**b**) The overview of the microfluidic SERS sensor with tubes inserted as the inlet and outlet for analyte transportation. Reproduced with permission from [84]. Copyright (2014) Wiley-VCH Vertag.

Mao et al. fabricated a SERS sensor consisting of a PDMS (polydimethylsiloxane) microchannel cap and a nanopillar forest on the basis of a SERS-enhancement substrate (Figure 9) [84]. The nanopillar forest was obtained via a new oxygen–plasma-stripping procedure and consisted of Si pillars covered by a thin layer (50 nm) of sputtered Ag. The reproducibility of the measurements was improved considerably by the flow cell method, compared with the results obtained by the traditional drop-casting of an analyte solution onto the open metallic surface, and could greatly reduce relative measurement errors. The sensor showed much higher measurement repeatability than the open substrate, and it reduced the sample preparation time from several hours to a few minutes, which makes the device more reliable and facile for tracing chemical and biological analysis.

**Figure 10:**
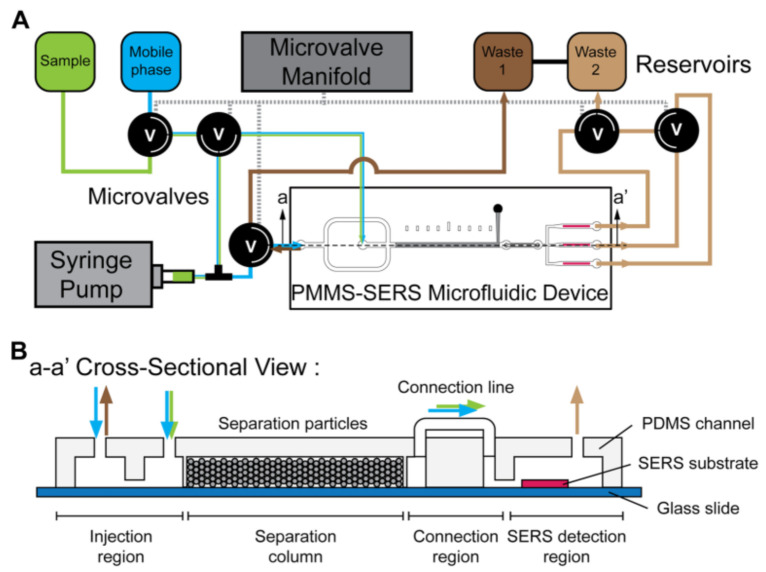
(**A**) Schematic diagram and (**B**) cross-section view of the particle-based microfluidic molecular separation (PMMS) SERS device. Reproduced with permission from [85]. Copyright (2019) Springer.

Wang et al. developed a particle-based microfluidic molecular separation (PMMS) integrating SERS sensing platform (Figure 10), which could separate complicate molecule mixture and achieve in situ SERS detection [85]. The platform consists of an automatic microfluidic control system to precisely control the sample and reagent flow in the PMMS SERS device, composed of a 5 μm particle-packed separation column followed by a two-dimensional Ag nanostructural substrate. This platform enables an automatic and sensitive purine derivatives analysis, which could be beneficial in applications requiring bacteria identification and quantification, such as environmental monitoring and drug development. 

#### 3.2.3. Microfluidics with Metallic Colloidal Nanoparticles for SERS Sensing 

**Figure 11:**
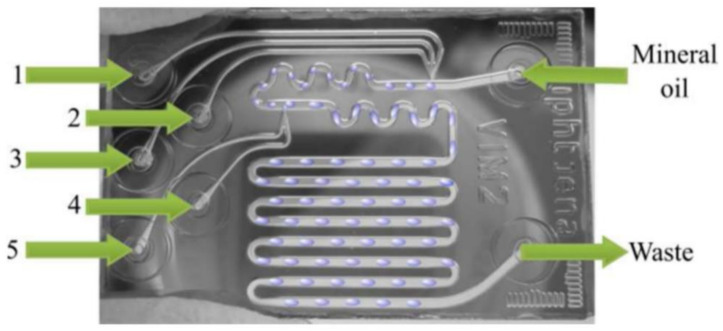
Droplet-based SERS microfluidics platform: ports 1–5 are used for the injection of sample, SERS-enhancement substrate, and their aggregation agent. Reproduced with permission from [86]. Copyright (2016) ACS publications.

Besides integrating the SERS substrate with microfluidics, there is another way to create a SERS-sensing mechanism by simultaneously loading colloidal nanoparticles with analytes into the microchannel. For example, Hidi et al. developed a glass droplet-based microfluidic chip (Figure 11) for the detection of nitroxoline (NTX) in human urine samples [86]. Firstly, the Ag NPs and NTX solutions were pumped via port 1 and port 3, respectively. T-junction can generate droplets in the continuous phase of oil efficiently. Then, the aggregation agent, potassium chloride solution (KCl), was injected into the already existing droplets via port 4. The mixing of substances was assured by the meandering channels, while a long measurement loop allowed fine-tuning of the measurement position. In this way, the in-droplet concentration of the analytes could be easily and automatically adjusted. This platform allows for high-throughput and multiplex detection and can avoid cross-contamination of samples [87]. 

Wu et al. developed a SERS-assisted 3D barcode microfluidic chip for a simultaneous, high-throughput, and multiplex immunoassay [88]. First, multiple proteins in different samples were spatially separated using a microfluidic patterned antibody substrate, forming a 2D hybridization array. Afterward, the mixture of SERS probes (Au and Ag nanorods) was flowed into the channel and captured by the corresponding antigens, forming a hybridization array used to identify and quantify the proteins. As different SERS probes were labeled with different Raman reporters, they were able to be employed as “SERS tags” to incorporate spectroscopic information into the 3D barcode. The microfluidic device with four reaction chambers was designed for the detection of the four different IgG biomarkers. Through integration with the microfluidics channel, one-step multiplex detection of human IgG, mouse IgG, and rabbit IgG within 30 min could be achieved with an ultra-sensitivity as low as 10 fg mL^−1^. Furthermore, in their later research [89], the authors successfully achieved multiplex detection of multiple breast cancer biomarkers, including carbohydrate antigen 15-3 (CA153), carbohydrate antigen 12-5 (CA125), and carcinoembryonic antigen (CEA) diagnosis. The system might be expected to provide multi-parallel high-throughput biomedical applications.

Although much work has been done to improve the mixing performance of the “injecting-nanoparticle” SERS microfluidic system, the external injection of nanoparticles seems a tedious process for microfluidic SERS applications. In addition, random colloidal nanoparticle aggregation is still a non-ignorable problem that affects the consistency of Raman signals. Thus, microfluidic systems coupled with solid surface-based substrates, featuring stable and ordered structures, have more potential for automatic detection in practical application.

### 3.3. Microfluidics with LSPR

#### 3.3.1. Microfluidics Integrating LSPR Sensor for Biomolecular Detection Only

**Figure 12:**
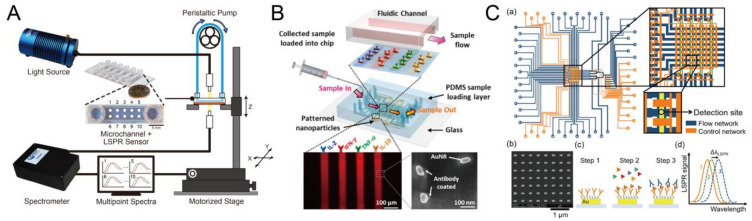
Biomolecular detection by optofluidic localized surface plasmon resonance (LSPR) device. (**A**) Schematic of the LSPR system consisting of a light source; spectrometer; peristaltic pump; LSPR sensor attached to a commercial microchannel device; and motorized stage for real-time, multi-point (10 spots) immunoglobulin G (IgG) detection. Reproduced with permission from [90]. Copyright (2017) MDPI. (**B**) The LSPR sensor consists of a PDMS microfluidic channel embedded with the multi-parallel AuNR (Au nanorod) array pattern coated on the glass substrate that can be used for multiplex cytokine detection. Reproduced with permission from [92]. Copyright (2016) ACS publications. (**C**) The quake valve-based microfluidic device integrating the gold nanorod array pattern is used for multiplex cytokine detection. Reproduced with permission from [93]. Copyright (2018) ACS publications.

Compared to other optical sensors, LSPR-based biosensors can be constructed in a relatively simple optical setup and minimum optical alignment. Therefore, it has been widely integrated with microfluidics for real-time, multi-parallel biomolecular monitoring in the laboratory settings or even in the point-of-care settings. For example, Lin et al. reported a multi-point analyte detection with a simple rapid thermal annealing (RTA)-fabricated LSPR sensor [90]. They optimized the LSPR sensor to the highest sensitivity around 189 nm RIU^−1^ by tuning gold deposition thickness and RTA temperature. After that, a commercial microchannel was integrated with the sensor for real-time immunoglobulin G (IgG) detection, as shown in Figure 12A. On the other hand, multiplex analyte detection is also a popular topic for researchers. Chen et al. proposed an LSPR sensor with 480 sensing spots integrating microfluidic array. A parallel and multiplex cytokine detection (IL-2, IL-4, IL-6, IL-10, IFN-γ, and TNF-α) in serum with high sensitivity of 6.46–20.56 pg/mL was demonstrated [91]. Furthermore, from the same group, Oh. et al. used this microfluidic design to detect off-chip T cell cytokine secretion profile [92], shown in Figure 12B. In essence, they used a syringe pump to load the secreted cytokine into the microfluidics. Then, the target analyte mentioned above was monitored. Yavas et al. designed dual-layer microfluidics to achieve multiplex cytokine detection [93]. Accordingly, the orthogonal microchannel was operated as a flow direction control, known as the quake valve. With the precise fluid control, sensitive LSPR sensor using amplification antibody, the researchers achieved an extremely low LOD, as shown in Figure 12C.

#### 3.3.2. Microfluidics Integrating LSPR Sensor for Cell Manipulation Followed by In Situ Biomolecular Detection 

**Figure 13:**
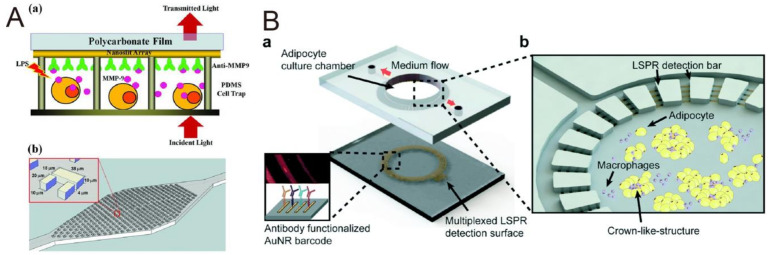
The microfluidic LSPR platform for on-chip cell manipulation followed by in situ biomolecular detection. (**A**) (a) Schematic of the device for matrix metalloproteinase 9 (MMP-9) detection. After cell stimulation, secreted-MMP-9 directly binds to the gold nanoslit array. Then, the amount of the MMP-9 secretion is measured as the transmission spectrum received. (b) The cell trapping microfluidics. Reproduced with permission from [94]. Copyright (2013) Wiley-VCH Vertag. (**B**) (a) Schematic of the adipocyte culture and LSPR sensing platform. The AuNR pattern coated on the glass substrate allows a multiplex cytokine measurement. The upper PDMS layer has a cylinder chamber for adipocyte culture. (b) The culture chamber surrounded by a block array provides a microenvironment for macrophage formation and can only allow the small secreted protein to pass through the gap for further LSPR sensing. Reproduced with permission from [95]. Copyright (2018) Royal Society of Chemistry.

Besides biomolecular detection, a microfluidic device can be applied for more advanced functions, such as cell separation, cell sorting, and creating a microenvironment for cell secretion detection. Therefore, many researchers have utilized such a device for investigating cell secretory activities. For instance, Wu et al. [94] proposed a microfluidic channel that contains an array of U-shaped cell traps for THP-1 cells trapping followed by lipopolysaccharide (LPS) stimulation. Then, after matrix metalloproteinase 9 (MMP-9) is secreted, an LSPR-based sensor can realize the long-term, real-time, in situ monitoring. As shown in Figure 13A, Zhu et al. demonstrated multiplex cytokine (IL-6, TNF-α, IL-10, and IL-4) detection with an LSPR sensor. Briefly, they bound a PDMS microfluidic device with a glass cover slide to construct a 4mm diameter cylindrical chamber for adipocytes and macrophages culture. Then, an LSPR sensor was bound temporarily for cytokine secretion detection after lipopolysaccharide (LPS) stimulation, as shown in Figure 13B. From their results, a rapid, sensitive, multiplex, and real-time assay was achieved. Moreover, the process of adipose tissue turning into the adipocyte was also observed [95].

### 3.4. Microfluidics with Photonic Crystal Sensors

**Figure 14:**
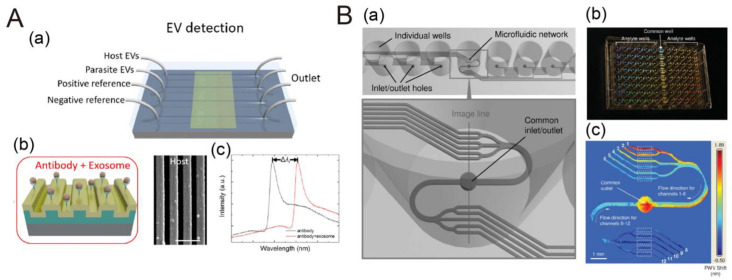
PC-based biosensors integrated with a microfluidic device. (**A**) (a) Schematic diagram of PC biosensor chips for extracellular vesicle (EV) detection. (**A**) (b) The mechanism for EV label-free detection and the SEM (scanning electron microscopic) image of host EVs immobilized on the PC surface. (**A**) (c) The binding of specific EV will affect resonant reflection and cause a spectral shift of Δλr. Reproduced with permission from [96]. Copyright (2018) ACS publications. (**B**) (a) Schematic of a 96-well microplate integrated with PC-based biosensors. (**B**) (b) Photograph of the bottom of the 96-well microplate incorporating microfluidic channel integrated with PC-based biosensors. (**B**) (c) Spatial peak wavelength value (PWV) shift image detected by 2D spatial image instrument that could be calculated by PWV shift values. Reproduced with permission from [98]. Copyright (2007) Royal Society of Chemistry.

PC-based sensors have been applied to biological and biochemical fields such as multiple biological target detection and biomolecular interaction analysis. Recently, analysis of membranous extracellular vesicles (EVs) such as exosome have been studied to diagnose various diseases [96]. The microfluidic PC-based biosensor only requires a small volume of sample reagents and can provide label-free, real-time analysis. Moreover, the measured time can be greatly reduced. An example is shown in Figure 14A. The surface of the PC-based biosensor was sequentially modified by polyvinylamine (PVA) and glutaraldehyde (GA). After that, CD63 antibody was also functionalized on the sensor surface to recognize CD63 presented on EVs. The changes in the refractive index caused by the recognition of EVs will be detected by PC-based biosensor. The LOD for the biosensor was 2.18 × 10^9^ EVs/mL with high specificity. The second example is integrating a 384-well microplate with a PC-based biosensor and a series of microfluidic channel for molecular kinetic reaction rate measurement [97]. The PC structure was fabricated with a plastic-based molding process that can be inexpensively manufactured. With advantages of microfluidics such as low reagent consumption and rapid response time, this study used the heparin and lactoferrin as examples, with the device being able to measure kinetic association and dissociation rate constants by detection of analyte binding. The sensor surface was sequentially modified by amine polymer and GA. Streptavidin was added to react with GA. Finally, biotinylated heparin was immobilized as a probe for lactoferrin to measure kinetic reaction rate. The same group also demonstrated IgG protein kinetic binding response measurement in a 96-well microfluidic and microplate-based platform integrated with PC-based biosensors (Figure 14B) [98]. Protein A was immobilized on sensor surface to recognize various IgG proteins in fluid. The change of peak wavelength value (PWV) was able to be be detected by a 2D spatial image instrument to analyze the kinetic interaction.

### 3.5. Microfluidics with Guided-Mode Resonator

**Figure 15:**
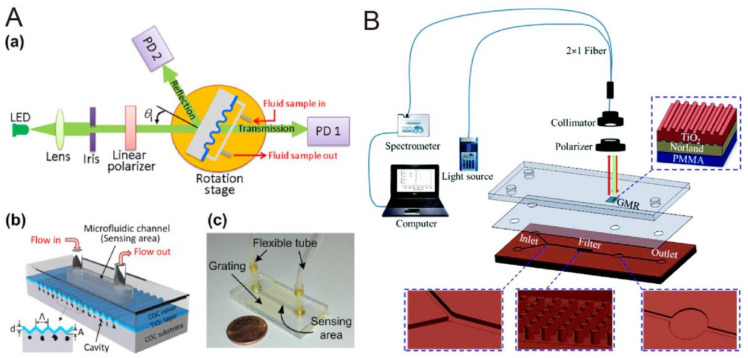
(**A**) (a) Schematic of the GMR optofluidic sensing system. (b) Schematic and (c) optical image of the disposable GMR biosensor chip. Reproduced with permission from [99]. Copyright (2017) Elsevier. (**B**) Schematic of the three-layer lab-on-chip system and optical read-out setup. Reproduced with permission from [100]. Copyright (2018) Royal Society of Chemistry.

Currently, several research groups are already integrating the GMR sensor into the microfluidic device to produce an optofluidic system in order to shorten the reaction time for biosensing, or even to provide the functionality of the sample process. Lin et al. developed an optofluidic system capable of monitoring the dinitrophenyl/anti-dinitrophenyl interaction [99]. Their GMR sensor was composed of a cyclic olefin copolymer substrate with 416 nm period, 100 nm amplitude 1D grating, and a 125 nm sputtered thick TiO_2_ layer for waveguiding. Instead of using white light source and spectrometry, the authors utilized a low-cost LED and a photodiode as the light source and detector, respectively, in order to lower the total cost of the system (Figure 15A-a). The sensor was then bonded to a cyclic olefin copolymer (COC)-based microfluidic channel to form an optofluidic device (Figure 15A-b). More importantly, they applied the lock-in technique at 1 kHz operation frequency to improve the SNR. From their result, the optofluidic system can achieve high refractive index (4.1 × 10^−5^ RIU^−1^) in reflection mode compared to other optical sensing techniques. Furthermore, they modified dinitrophenyl onto the TiO_2_ surface to perform anti-dinitrophenyl biosensing. The LOD was 75 ng/mL with 0.9858 coefficient of variance, indicating high linearity and high sensitivity of the system. To enable POC, the direct cellular or biomolecular analysis of a real clinical sample will be a huge obstacle due to its complexity and variation between different patients. Hence, the microfluidic-based sample process becomes a critical module for system integration. Taking blood as an example, the existence of blood cells and debris will affect optical sensing by generating extra absorption and scattered light. In order to eliminate the interference, the plasma should be extracted first by removing all the cells and debris. On the basis of this concept, Tsai et al. proposed a microfluidic with a blood process module integrating GMR sensor for diluted blood sample analysis [100]. The microfluidic device can be divided into two regions—filter region and sensing region. In the filter region, a micropost array was fabricated to extract the plasma from 10,000x diluted blood by filtering the cells. In sensing region, a GMR sensor, composed of PMMA substrate, an optical adhesive grating with 555.5 nm period/110 nm depth, and a TiO_2_ layer, was integrated by a double-sided tape. The device was reported to have 186 nm/RIU sensitivity from the sucrose solution. The device then performed biosensing of C-reactive protein and the corresponding limit of detection was 3.2 ng/mL with approximately five orders of dynamic range. Compared to ELISA (enzyme-linked immunosorbent assay) kit, their device had higher LOD but wider dynamic range. Most importantly, they introduced rat blood to the microfluidic device for C-reactive protein (CRP) on-chip sensing and compared the results of direct sensing from plasma or blood. In terms of the peak shift, the shift of on-chip sensing was identical to that of direct sensing from plasma. However, the shift of direct sensing from whole blood was shown to have higher peak shift due to the interference of blood cells and debris. 

## 4. Application of Integrated Sensors to Point-of-Care 

### 4.1. Review of Integrated Biosensors and Future Trend 

The aforementioned optical biosensors integrated with various technologies have the potential to provide more accurate and more comprehensive diagnoses of diseases. For further POCT applications, it is necessary that the integrated optical sensors are low-cost, have rapid detection, and are simple to operate when migrating from laboratory tests to near the patient diagnosis. 

There are new or evolving technologies that have the potential to disrupt the established POC diagnosis. In our opinion, recent optical biosensor development for POCT can be categorized in the following ways:

- Lab-on-a-chip: Microfluidic techniques have been extensively employed for POC biosensors. The integration of microfluidic and optical techniques as elaborated in the previous sections incorporates several sensing functions in a chip, in addition to target analyte delivery, thus simplifying the steps of testing and enabling the miniaturization of biomedical devices [101].
- Surface tension or evaporation-induced flow: Since the sample volume can be greatly reduced in the microfluidic devices, the flow driven force can be generated by surface tension or evaporation driven flow, which can eliminate the requirement of external pump or further enrich targeted molecules to achieve a higher sensitivity. For example, Kumar et al. demonstrated a surface tension-driven flow to guide 4-mercaptopyridine into a suspended plasmonic nanohole array for SERS detection [102]. Regarding to the molecule concentration, one example involves the evaporation-induced spontaneous flow of 100 pL inkjet-printed droplets on the photonic crystal biosilia to perform SERS detection [103]. Another method is using the pyro-dispensing technique to guide and concentrate targeted molecules into the fluorescence-based sensing substrate. [104].
- Lateral flow and vertical flow immunoassays: Lateral flow immunoassay (LFI) is one of the most mature POC technologies [105]. Examples of lateral flow tests include pregnancy, infectious diseases, cancer, cardiac diseases, illicit drug abuse, and influenza tests [106,107]. The alternative to LFIA is vertical flow immunoassay (VFI), which offers several key advantages, including faster analysis time and the absence of a false-negative inducing hook effect [108]. The VFI has been explored to detect antibody in human serum and bio-threat pathogens, among others [109,110].
- Wearable and continuous monitoring: Continuous monitoring of chronic disease and wellness is one of the most significant advances of POCT. For example, the Apple watch, a wearable device, uses green LED lights paired with light-sensitive photodiodes to detect the amount of blood flowing through the wrist at any given moment. The heart rate and electrocardiogram (ECG) are monitored [111]. Another example is for diabetes, wherein many commercial devices focus on continuous glucose monitoring (CGM) in various body fluids [112].
- DNA sequencing: DNA sequencing has become indispensable for basic biological research, biotechnology, and medical diagnosis. In recent years, DNA sequencing has been employed in the medical facility to determine if there is risk of genetic diseases in patients. Health analysts have predicted that DNA sequencing will shift from a laboratory-based analysis to POC performed by the patients off-site within the next five years [113].

A brief summary of integrated optical biosensors in recent years for POC applications is listed in Table 1. They are mainly, but not all, based on the microfluidic platform. We believe in the near future there will be more optical biosensors integrated with technologies beyond microfluidic channels for POCT.

### 4.2. Market and Future Perspectives

Depending on the definition of the POC market, the estimation of the overall market size of POC varies but was generally reckoned to be more than USD 20 billion in 2018, with a CAGR (compound annual growth rate) within the next 5 years of around 10% [121,122,123]. The pregnancy test and CGM led the POCT market in recent years but their growth has been saturated. The awareness of healthcare and disease prevalence in developed countries are the driving forces for the growth of the POCT market [124]. In developing countries such as China and India, the demand of POCT is growing at a faster pace as more of the population enters the middle class and increases the demand for a high standard of healthcare [124]. For rural areas where medical resources are limited, POCT plays a critical role in timely availability of medical diagnosis. To satisfy the demand on detecting multiple diseases with high accuracy, we anticipate that integrated biosensors will be the future trend of POCT.

## 5. Conclusions

In this article, we overviewed optical biosensing technologies. Some, such as SPR, fluorescence, and Bragg grating are commercially available, while others are currently only found in laboratory testing. The rapid growth of POCT changes the landscape of biosensor development and future business opportunities. We envision that there are more integrated optical biosensors for POC. The integrated optical biosensors have many advantages. First, the integration miniaturizes the components that construct optical biosensors. Second, the sensitivities and accuracy can be further improved by incorporating other physical, mechanical, and electrical sensing technologies. Many optical biosensors facilitate microfluidic for target analyte delivery, miniaturization, and performance improvement. On the other hand, the integration of optical biosensors with field-effect electrical charge sensing is rare. We expect there will be more optical biosensors that simultaneously extract the electrical charge properties of the target analyte on the basis of semiconductor fabrication. The integrated optical biosensors are for the benefit of POC. Future perspectives in the development of biosensors, in our opinion, are on the continuous improvement of lab-on-a-chip, LFI and VFI, wearable and continuous-monitoring, and DNA sequencing. Optical biosensors will play an indispensable role in future POCT by integrating with microfluidic, mechanical, and electrical biosensing technologies.

## Figures and Tables

**Table 1:** Review of optical biosensors with various sensing technologies for point-of-care (POC).

Sensor Type	Detector Technology	Target Analyte	Reference
Fluorescent	Microfluidic and FRET	Noroviruses (NoV)	[16]
	Microfluidic and loop-mediated isothermal amplification (LAMP)	*Neisseria meningitidis*	[114]
Colorimetric	ELISA and cell phone/charge-coupled device (CCD)	Ovarian cancer HE4 biomarker	[17]
	USB interface mobile platform, microfluidic and ELISA	BDE-47	[115]
SPR	Microfluidic and SPR	*Escherichia coli* *Staphylococcus aureus*	[18]
	SAW (surface acoustic wave), microfluidic and SPR	Avidin-biotin binding	[116]
	SAW and SPR	AFB1	[117]
	LSPR and microfluidic	Protein binding	[118]
GMR	GMR, microfluidic and a CMOS (complementary metal oxide semiconductor) camera	IgG	[19]
PC	PC and microfluidic	CD40 ligand antibody EGF antibody Streptavidin Thrombin	[119]
SERS	SERS and microfluidic	Hepatitis B virus antigen	[20]
	SERS and microfluidic	Rabbit IgG protein	[120]
